# Genomic-assisted refinement of heterotic groups in short-duration maturing tropical yellow and orange maize inbred lines

**DOI:** 10.1186/s44399-026-00032-2

**Published:** 2026-03-04

**Authors:** Idris Ishola Adejumobi, Wende Mengesha, Melaku Gedil, Silvestro Meseka, Adamu Masari Abubakar, Odette Tegawende Bonkoungou, Fatoumata Ouattara, Baffour Badu-Apraku, Abebe Menkir, John Derera, Hapson Mushoriwa

**Affiliations:** 1 International Institute of Tropical Agriculture, Ibadan, PMB 5320, 200001 Nigeria; 2 Department of Plant Science, Amadu Bello University, Zaria, Nigeria; 3 Institut de l’Environnement et de Recherches Agricoles (INERA), 01 B.P.910, Bobo-Dioulasso 01, Burkina Faso

**Keywords:** Admixture analysis, DArTag SNP panel, Discriminant analysis, Diversity analysis, Phylogenetic analysis, Population structure, Heterotic group

## Abstract

**Background:**

Understanding the genetic diversity and population structure of maize inbred lines is fundamental for effective hybrid development and strategic heterosis exploitation. The objective of this study was to refine and validate heterotic groups within a diverse collection of extra-early and early maturing tropical yellow and orange maize inbred lines. A total of 1,437 elite tropical yellow and orange maize inbred lines spanning early and extra-early maturing groups were genotyped using a panel of 3,305 DArTag SNP markers. In addition, the study evaluated 280 hybrids comprising 276 hybrids (from 214 inbred lines crossed with four standard testers) and four commercial checks. The yield performance trials used a 14 × 20 alpha-lattice design with two replicates over two years.

**Results:**

Marker-based diversity analysis revealed average marker polymorphism of 0.328, minor allele frequency of 0.265, and expected heterozygosity of 0.315. Principal component analysis (PCA), discriminant analysis of principal components (DAPC), and ancestral admixture analysis revealed three genetic clusters, while phylogenetic analysis supported the existence of two major clusters with groups one and three from DAPC and admixture methods combined into a single phylogenetic group. Several sub-groups were identified within the main genetic pools and maturity rather than kernel color was the major driver of these sub-groups. For operational ease in breeding applications, the lines were assigned to two major heterotic groups. The average performance of the hybrid for grain yield and heterosis estimates from between maturity-based heterotic subgroups were consistently and significantly (*p* < 0.001) higher than those from within maturity-based heterotic subgroups.

**Conclusion:**

These results provide a robust framework for parental line selection, heterotic grouping, and hybrid breeding strategies, facilitating the development of extra-early and early superior yellow/orange maize hybrids adapted to tropical environments.

**Supplementary Information:**

The online version contains supplementary material available at 10.1186/s44399-026-00032-2.

## Introduction

Maize (*Zea mays* L.) stands as a cornerstone of global food security, particularly in tropical and subtropical regions where it serves as a primary staple food and a vital source of income for millions of smallholder farmers. Within these diverse agro-ecologies, the demand for extra-early and early maturity maize varieties, known as short-duration maturity, spanning between 80 and 99 days, is increasing due to their critical role, particularly in short-duration rainfall zones [1, 2]. Additionally, in regions with long durations of rainfall, this class of varieties enables multiple cropping cycles and facilitates crop intensification [3]. Concurrently, consumer and industrial preferences for specific kernel types, notably yellow and orange maize, are growing. This is driven by nutritional benefits such as provitamin A content notably in orange kernels for provitamin A, and in industrial applications, particularly in the livestock feed industry [4–6]. Developing high-yielding hybrids that simultaneously meet these maturity and kernel colour demands for smallholder farmers in sub-Saharan Africa (SSA) is a key objective for the Maize Improvement Programmes (MIP) of the International Institute of Tropical Agriculture (IITA).

The success of hybrid breeding programmes hinges significantly on the efficient exploitation of heterosis, otherwise called hybrid vigour. This is conventionally achieved through the strategic formation of heterotic groups. Heterotic groups are genetically distinct pools of inbred lines that, when intercrossed, consistently produce superior hybrids [7]. The systematic assignment of inbred lines to specific heterotic groups streamlines breeding efforts by guiding parental selection, optimizing crossing schemes, and maximizing the genetic gain from heterosis [8]. Historically, heterotic groups in the IITA-MIP short-duration maturity germplasm have been established through empirical testcross evaluation and pedigree analysis, often resulting in a limited number of broadly defined groups [9–11].

Defining and refining heterotic groups in maize germplasm is fraught with unique challenges. The high genetic diversity inherent in the populations, coupled with complex population structures and the often-limited phenotypic evaluation of all possible hybrid combinations, can lead to imprecise or sub-optimal grouping based solely on conventional methods [12]. Furthermore, the specific requirements of extra-early and early maturity and distinct kernel colour types may introduce additional layers of complexity, as these traits might be associated with particular genetic backgrounds or heterotic patterns that are not always evident from phenotypic data alone. The empirical nature of traditional grouping can be time-consuming and resource-intensive, particularly in large breeding programs like the Maize Improvement Program (MIP) of IITA dealing with thousands of new or elite inbred lines.

The advent of high-throughput molecular marker technologies, such as DArTag SNP markers, offers an unprecedented opportunity to address these limitations. Genomic data provides a comprehensive and unbiased assessment of genetic relationships, population structure, and genetic diversity at a much higher resolution than pedigree or phenotypic data alone [13]. By leveraging thousands of genome-wide markers, it is possible to precisely quantify genetic distances, delineate distinct genetic clusters, and infer ancestral contributions, thereby providing a robust foundation for genomic-assisted refinement of heterotic groups [14]. This molecular approach can complement and validate existing empirical groupings, reveal novel heterotic patterns, and facilitate the more accurate assignment of elite inbred lines to appropriate heterotic pools from a broader perspective.

Building on this foundation, we proposed two specific, testable hypotheses to guide our investigation and validate the utility of molecular data in our breeding program. The first being that the genomic clustering of tropical maize inbred lines will align strongly with existing maturity groups (extra-early and early) and with kernel color (yellow and orange), reflecting the historical breeding emphasis on adaptation. The second hypothesis is that the genomic-assisted heterotic groups will be robust and consistent across multiple clustering methods (DAPC, Admixture, Phylogeny), and that crosses between these refined heterotic groups and subgroups will yield significantly higher heterosis and superior grain yield compared to crosses made within the same groups.

This study aims to leverage a medium-scale DArTag SNP dataset to refine and validate heterotic groups within a diverse collection of extra-early and early maturity tropical yellow and orange maize inbred lines. Specifically, the study investigated the underlying genetic structure of these inbreds, assessed their genetic relationships, and utilized genomic information to propose an optimized framework for heterotic grouping. The insights gained will enhance the efficiency of hybrid breeding strategies, accelerate the development of breakthrough product (high-performing, market-tailored hybrid maize), and ultimately contribute to improved food security in tropical environments.

## Materials and methods

### Plant material

The study utilized a panel of 1,437 diverse tropical maize inbred lines, sourced from the MIP of IITA. These lines comprised 788 and 649 inbred lines of extra-early and early maturity groups, respectively, representing key adaptation traits for diverse agro-ecologies in SSA. The panel of inbred lines also encompassed distinct yellow and orange kernel color types within each maturity group, reflecting important nutritional and market preferences in the region. The 1,437 inbred lines were derived from 23 source populations (15 broad-based and 8 narrow-based) that began from S_1_ line development. Badu-Apraku and Fakorede [15] reported the details on how these source populations were developed. For the line development, the S_1_ lines were advanced to S_4_ stages through inbreeding, with each cycle of selfing being followed by field evaluation under artificial *Striga hermonthica* infestation and induced drought stress before selection. At the S_4_ stage of inbreeding, combining ability was assessed by crossing selected lines (250–300) to broad-based testers. Based on the testcrosses’ performance, about 100 S_4_ lines were advanced through inbreeding to the S_7_ stage, where homozygosity of the lines is expected to be 99%. The number of inbred lines and the characteristics of each source population were presented in Table 1 while the detailed pedigree information is presented in Table S1.

### Evaluation of hybrids developed from between and within tester groups

To validate potential heterotic groupings, eight standard testers, namely TZEI 17 and TZEI 13 (early yellow tester), TZEIOR 164 and TZEIOR 108 (early orange tester), TZEEI 58 and TZEEI 79 (extra-early yellow testers), and TZEEIOR 97 and TZEEIOR 197 (extra-early orange testers) kernels were included among the genetic materials. We used a subset of the 1,437 inbred lines, i.e. 214 inbred lines comprising 81 extra-early and 133 early maturing inbreds to generate 276 testcross hybrids. These include within-cluster hybrids generated from crossing testers to inbred lines within the same heterotic group (extra-early – 44 hybrids, early – 80 hybrids) and between-cluster hybrids resulting from crossing testers to inbred lines outside their respective heterotic groups (extra-early – 48 hybrids, early 104 hybrids).

**Table 1:** Summarized information on the panel of inbred lines focusing on kernel color, number of inbred lines, and percentage of total panel

Maturity Type	Kernel Color	Number of Inbred Lines	Percentage of Total Panel (%)
Early	Yellow	452	31.45
Early	Orange	196	13.64
Extra-Early	Yellow	200	13.92
Extra-Early	Orange	589	40.99
Total		1,437	100.00

The testers selected for inclusion in this study were previously reported to belong to different heterotic groups. For the early yellow and orange testers (TZEI 17, TZEI 13, TZEEIOR 164, TZEEIOR 108) [16] and for extra-early yellow and orange testers [2, 17]. The 280 genotypes comprising 276 testcross hybrids and four commercial checks namely SAMMAZ 41, SAMMAZ 56, Sosani, and WAC58PVEE, were evaluated for two years at Ikenne IITA sub-station. Ikenne sub-station (7°52′ N, 30°44′ E, 60 m altitude) is in the lowland humid forest Zone. The sub-station is characterized by strongly leached, highly weathered, and well-drained loamy sand eutric nitisols (FAO/UNESCO, 1994) with flat and uniform terrain. The rainfall in the southwestern part of Nigeria where Ikenne is located is relatively high with a bimodal pattern (average annual total of 1732 m) spanning from April to October. Field trials were conducted using a 14 × 20 alpha-lattice design with two replications. Plot size was single-row plot of 3 m spaced at 0.4 by 0.75 m. Three seeds were sown at planting, thinned to two plants/hill after establishment, and stand count was based on this density giving a population of 66,667 plant/ha. We followed all standard agronomic practices to ensure optimal plant growth and minimize non-experimental variation.

### Phenotypic data collection

Key agronomic traits were assessed for data collection from the hybrid trials. These include grain yield measured in kilogram from harvested plots. Days to 50% anthesis (DA) recorded as number of days from planting to when 50% of plants in a plot have shed pollen. Days to 50% silking (DS) recorded as number of days from planting to when 50% of plants in a plot have extruded silks. Anthesis-Silking Interval (ASI) as DS – DA. Plant and ear height (cm) is the distance from the ground to the base of the tassel and uppermost ear, respectively. Plant and ear aspect (1–9 scale) as visual rating of plant and ear health, fill, and disease/pest damage (1 = excellent, 9 = poor).

### Leaf sampling and genotyping

Advanced inbred lines (S7 stage) were planted in the field to obtain leaf samples for genotyping. Approximately three weeks after planting, we carefully sampled young and fully expanded leaf tissue from the developing seedlings. Precisely, we punched small leaf discs from the three-week-old seedling leaf samples. These discs were meticulously placed into individually labeled deep-well plates for efficient processing. The leaf samples were collected over dried ice on the field and immediately transferred to -80 degrees Celsius on arrival at the IITA Bioscience laboratory to preserve the integrity of the DNA. Samples were stored under this condition for 72 h after which the lyophilization process using a Labconco Freezone 2.5 L System (Marshall Scientific, USA) was used to remove moisture. The lyophilized leaf samples were then shipped to Intertek Laboratory in Australia for mid-density DArTag genotyping with 3,305 SNP markers following the procedure described by Kilian et al. [18].

### Genotyping data quality control and processing

Upon receiving the genotyping results containing 3,305 SNP marker sequence information across the 10 chromosomes of the maize genome, rigorous quality control (QC) involving a two-step approach was applied to the raw SNP data from the panel of inbred lines. Before this two-step approach was implemented, the data were converted to VCF format in TASSEL [19] for suitability of QC operation in Plink 2.0 [20]. Following importation in to Plink, the first step was to eliminate SNP markers with poor call rate (< 80%), genotype with high missing data points (> 20%), and markers with high heterozygosity (> 20%). The first step operation resulted in the elimination of 528 markers leaving 2,777 SNPs as intermediate dataset for further QC implementation. We then applied a stringent value of 5% MAF to the intermediate dataset (2,777 SNPs) to obtain a high-quality dataset (2,092 SNPs) for downstream analyses reported in this study and further usage (Genomic prediction) in the maize program. To address the suitability of markers for population structure analysis, we determined that an explicit Linkage Disequilibrium (LD) pruning step was not necessary. The DArTag panel markers are already relatively dispersed across the maize genome with low localized redundancy. Additionally, the subsequent clustering method, DAPC, is based on principal component analysis and is robust to markers in high LD. The missing genotypes in the 2,092 SNPs (final filtered) dataset were imputed using a k-nearest neighbor (kNN) approach implemented in the rrBLUP package [21]. The final filtered and imputed data was converted into a numerical genotype matrix in GAPIT [22], with individuals as rows and markers as columns.

### Statistical and bioinformatics analyses

All statistical analyses were performed using PLINK 2.0 [20] and the R programming language [23].

### Genetic diversity and population structure

The metrics (PIC, OH, and EH) were calculated on the 2,777 SNPs. This set represents the markers remaining after the initial, non-negotiable quality control steps (call rate, genotype missingness, and heterozygosity) were applied to the raw 3,305 SNPs. Crucially; this calculation was performed before applying the stringent 5% MAF threshold. The final 2,092 SNPs were used only to report the minimum, maximum, and mean minor allele frequency (MAF). This approach ensures that the reported diversity metrics reflect the full spectrum of genetic variation present in the reliable germplasm panel, while the MAF statistics validate the quality of the markers used for downstream work. The genetic diversity and population structure of the 1,437 inbred lines were investigated using multiple approaches. The first approach was Principal Component Analysis (PCA) using the PCA function in the FactoMineR package [24]. The PCA visually summarized the major patterns of genetic variation among the inbred lines, with individuals grouped by kernel color and maturity type. Model-based Clustering using ancestral admixture analysis in LEA package [25] was the second approach. In this package, the sparse non-negative matrix factorization (snmf) function was used with the default alpha (Dirichlet prior) of 0.5 for all tested K values. This value allows for a moderate degree of expected admixture. The optimal number of ancestral populations (K) was determined by evaluating cross-validation error or likelihood values across a range of K values (1 to 20). The resulting Q-matrix was used to assign individuals to genomically pure clusters using a 70% membership coefficient threshold. Individuals with less than 70% membership were delineated as admixed lines and were retained as a separate group for visualization, but were excluded from subsequent analyses that required distinct, unambiguous cluster assignment such as initial definition of heterotic groups. The third approach was Discriminant Analysis of Principal Components (DAPC) using the adegenet package [26] to confirm the distinctness of the identified genomic clusters. For DAPC, the optimal number of genetic clusters (K) was first determined by using the “find.clusters” function with a maximum of 40 clusters. The number of PCs retained for the BIC calculation was determined using cross-validation to be 150 PCs, which ensured capture of the underlying genetic variance without introducing excess noise. The final DAPC model was then executed by retaining 200 PCs (accounting for approximately 85% of the total genetic variance) and K − 1 discriminant functions to visualize the structure. The phylogenetic analysis that unravels the evolutionary history and relationships among a group of organisms or genetic sequences was the last approach. This distance-based method necessarily partitions all 1,437 individuals (including admixed lines) into discrete branches based on genetic distance (GD – Gower dissimilarity matrix) between inbreds that was computed using the daisy function in the Cluster package [27]. The resulting phylogeny tree was constructed using the Ward.D2 method in ape package [28]. The phylogeny was used to visually confirm the major patterns of genetic divergence observed in the PCA and LEA results, and was overlaid with both kernel color and maturity group using the ggtree function in the ggtree package [29].

### Heterotic group delineation and refinement

We defined the genomic-based heterotic groups by integrating the results from ancestral admixture and DAPC with the phylogenetic analysis. In ancestral admixture and DAPC, we assigned the inbred lines to a group if their membership coefficient to a single ancestral population was above the defined threshold (≥ 70%). We carefully considered the individuals with mixed ancestry (admixed) for assignment based on their dominant ancestry for comparison of different methods. In addition, the phylogenetic analysis was also used to group inbreds into groups based on identified optimum K values revealed by the Elbow method in the NbClust package [30]. The heterotic classifications from these three methods were compared for concordance in a bar plot representation using the ggplot2 package [31]. Finally, we examined the distribution by kernel color (yellow/orange) and maturity class (extra-early/early) within the proposed genomic groups for practical breeding utility.

### Validation of heterotic groups

To empirically validate the usefulness of the genomic-based heterotic groups identified within the IITA-MIP germplasm, the performance of the 280 hybrids comprising 276 testcross and four hybrid checks was carefully evaluated. The mixed linear model was used to conduct analysis of variance (ANOVA) using the lmerTest package [32] in R following the model below:

$$\eqalign{{Y_{ijkl}} = {\rm{ }} & \mu {\rm{ }} + {\rm{ }}Re{p_j} + {\rm{ }}Rep{\rm{ }}{\left( {Blk} \right)_{j(k)}} + {\rm{ }}Hy{b_i} \cr & + {\rm{ }}Yea{r_l} + {\rm{ }}{\left( {Hyb{\rm{ }} \times {\rm{ }}Year} \right)_{il}} + {\rm{ }}{e_{ijkl}} \cr} $$

*Where; Y*_*ijk*_*is the phenotypic performance of hybrids for traits under consideration*,* µ is the average hybrid performance*,* Hyb*_*i*_*is the effect of hybrid i*,* Repj is the effect of replication j*,* Rep(Blk)*_*j(k)*_*is the block k effect nested in replication j*,* Year*_*l*_*is the effect of year l*,* Hyb × Year*_*(il)*_*is the effect of the hybrid i by year l interaction*,* and e*_*rrorijkl*_*is the residual effect.*

For the analysis, we considered the effects of hybrid, year, and the hybrid by year interaction as fixed effects to calculate the Best Linear Unbiased Estimates (BLUEs). We also considered Replication and Block nested within Replication as random effects to maximize precision by controlling for the field design variability. These BLUEs served as the basis for comparing grain yield performance and heterosis. We statistically compare the mean grain yield of hybrids resulting from inter-group crosses (i.e., crosses between parents belonging to different genomic heterotic groups) to those from intra-group crosses (i.e., crosses between parents from the same genomic heterotic group). This comparison was visually represented using box plots and statistically evaluated using the Wilcoxon rank-sum test, implemented in STATS package [33]. To maintain statistical rigor and control the Type I error rate across multiple traits, all p-values resulting from the Wilcoxon rank-sum tests were adjusted using the False Discovery Rate (FDR) method [34]. Additionally, the mean comparison figures and tables are supported by 95% Confidence Intervals (CIs) derived from the ANOVA/BLUEs estimation. Furthermore, the average standard heterosis (heterosis over the best performing commercial check) was calculated for both inter- and intra-group hybrids. These average heterosis values were then compared using the same statistical approach (Wilcoxon rank-sum test) to determine if crossing between the newly defined genomic heterotic groups consistently resulted in superior hybrid vigor compared to within-group crosses.

$$\eqalign{& {\rm{Standard}}\,{\rm{Heterosis}}\,\left( \% \right) \cr & = {\matrix{\,\,\,\,\,\,\,\,\,\,\,\,\,\,\,\,GY\,of\,hybrid \hfill \cr - GY\,of\,best\,commercial\,check \hfill \cr} \over \matrix{\,\,\,\,\,\,\,\,GY\,of\,best \hfill \cr \,commercial\,check \hfill \cr} } \times 100\% \cr} $$

Where; GY is grain yield in kg/ha.

The detailed information on the software and R-based packages used for conducting the statistical analysis is presented in Table 2.

**Table 2:** List of the software and R-based packages used in statistical analyses

Analysis Step	Software/Package	Version	Key Parameters/Thresholds
Data QC	PLINK/TASSEL	2.0/5.2.96	Call Rate < 80%, Het > 20%, MAF < 5%
Population Structure (LEA)	LEA (snmf)	3.18.0	K = 1 − 20, α = 0.5 (Default), 70% membership threshold
Population Structure (DAPC)	adegenet	2.1.11	BIC: 150 PCs; Final Model: 200 PCs, K − 1 discriminant functions
Phylogeny	Cluster/ APE	2.1.8.1/5.8-1	GD (Gower), Ward.D2 method
Mixed Linear Model (ANOVA/BLUEs	lmerTest/Lme4	3.1-3/1.1–37	Hybrid, Year, Hyb×Year (Fixed); Rep, Block (Random)
Mean Comparison	STATS	4.4.1	Wilcoxon rank-sum, FDR adjustment
Agreement between heterotic grouping methods	ggplot2	4.0.0	Bar plots

## Results

### Diversity indices of SNP markers

The diversity indices of the 2,092 high-quality SNP markers revealed an average PIC of 0.328, with a range of 0.003 to 0.500, an average of 0.265 with a range of 0.05 to 0.500 for MAF. The observed and expected heterozygosity averaged 0.032 (range: 0.000 to 0.206) and 0.315 (range: 0.000 to 0.500), respectively (Table S2).

### Population structure and genetic diversity

**Fig. 1:**
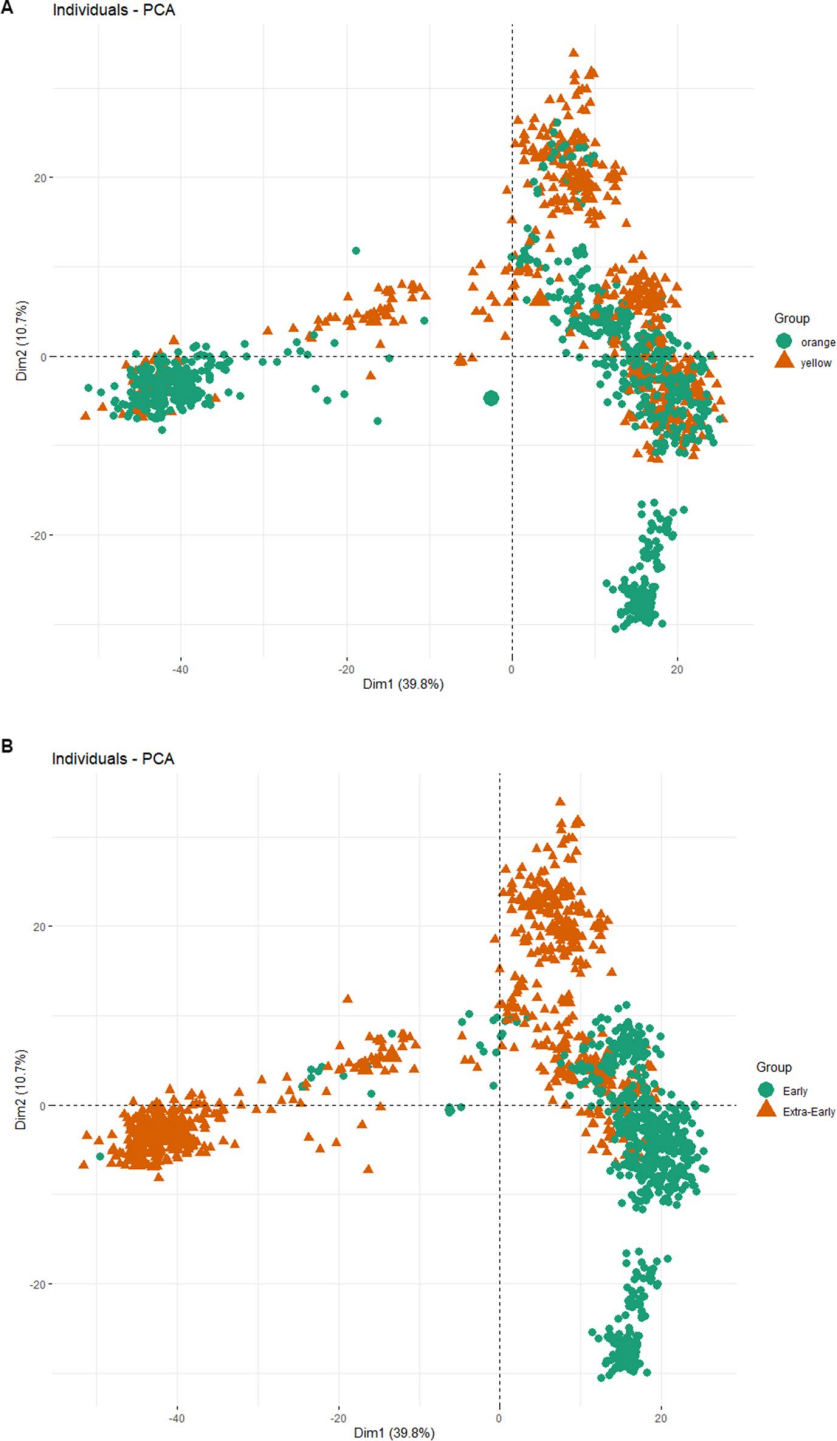
Visualization of population stratification overlaid by (**A**) kernel color and (**B**) maturity group from principal coordinate analysis

The analysis of 1437 elite yellow and orange inbred lines from extra-early and early maturity groups was performed using different methods. The first method (PCA) visually summarized the main patterns of genetic variation among the inbred lines into three genetic groups (Fig. S1). In the PCA plot, the first and second dimensions captured 50.5% of the total variation. When the plot was overlaid with kernel color, there was no clear distinction of group identity based on kernel color. The PCA visualization showed potential admixture and shared ancestry between several yellow and orange lines (Fig. 1A). However, overlaying maturity groups produced different results. The PCA analysis clearly separated early and extra-early maturity inbred lines. Along the first principal component (Dim1), which explains 39.8% of the genetic variation, there is a strong separation between the two groups. On this axis, the extra-early inbreds were dispersed into distinct clusters on the far left, the central region, and the top right of the plot. In contrast, early maturity lines were primarily grouped on the right side of Dim1 while showing a tighter grouping along the second component (Dim2). This dimension accounted for 10.7% of the variation. Some overlap exists between the two groups near the central axis, suggesting shared ancestry or gene flow between early and extra-early lines. The visualization supports the idea that maturity classification aligns with genetic structure (Fig. 1A). The other approach, Principal Coordinate Analysis, produced a similar visualization pattern (Sup. Figure 2 A and B).

**Fig. 2:**
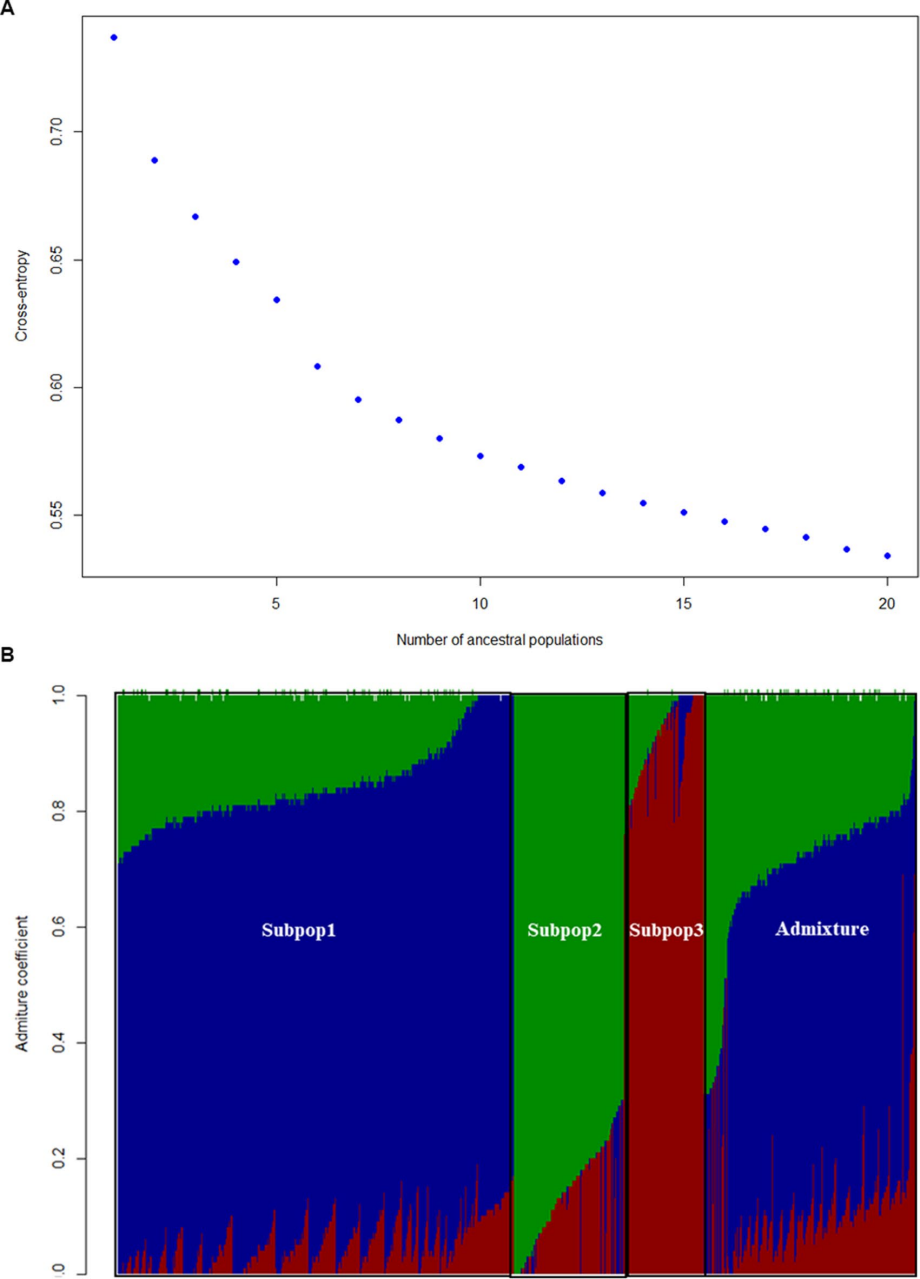
Population structure as revealed by admixture ancestry analysis. (**A**) Graph of cross-entropy by number of ancestral populations (**B**) Visualization of subpopulations 1 (blue), 2 (green), 3 (red), and admixed lines (admixture) defined by membership probability of 0.70

The ancestral admixture analysis that uses a model-based clustering approach revealed the presence of three main ancestral populations (K = 3), as shown in the cross-entropy plot (Fig. 2A). The cross-entropy values drop sharply up to K = 3 and begin to plateau, an indication that three ancestral groups best explain the genetic structure of the 1,437 inbred lines. The bar plot (Fig. 2B) shows the proportion of genetic ancestry in each individual across the three inferred subpopulations. Subpopulations one, two, and three exhibit distinct genetic compositions predominantly represented by blue (714 inbred lines), red (198 inbred lines), and green (145 inbred lines) components, respectively (≥ 70% membership probability). Similar to the results from PCA when we used kernel color as a cofactor, it does not align with the three genetic groups. However, based on maturity group, each of the inferred groups has unique characteristics — subpopulation one has 95% of the assigned members as extra-early inbred lines, subpopulation two has all members as early inbreds, and subpopulation three has all members as extra-early inbreds (Table S3). This pattern mirrors the broad dispersion of the extra-early inbred lines in the PCA plot. A substantial portion (380 inbred lines equivalence of 26%) of the lines also display mixed ancestry, indicating substantial levels of admixture and gene flow among the subpopulations. Among the eight inbred testers included in the inbred germplasm, we identified two testers as admixture (TZEI 17 and TZEEIOR 197) (Table S3).

**Fig. 3:**
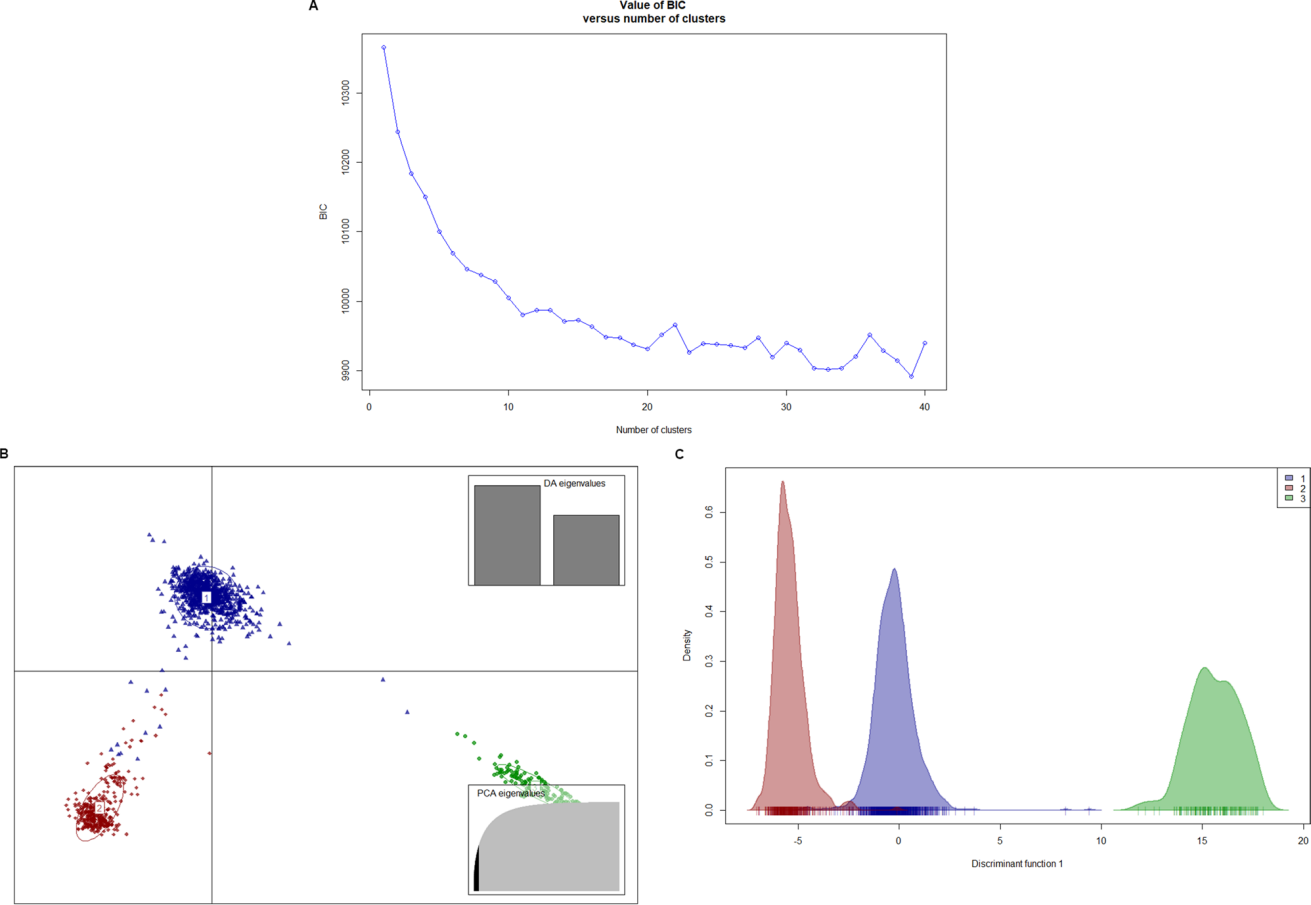
Population structure as revealed by discriminant analysis of principal component. (**A**) Graph of BIC vs. number of cluster (K) from where optimal K was inferred, (**B**) visualization of the three distinct genetic groups 1, 2, 3 (blue, red, and green, respectively) characterizing the 1,437 population of inbred lines, (**C**) density plot of the three genetic groups 1, 2, 3 (blue, red, and green, respectively) showing peaks with high genetic differentiation

To confirm and further refine the population structure observed in the PCA and ancestral admixture analysis, we performed the discriminant analysis of principal components. This analysis was based on the optimal number of clusters (K), determined by the Bayesian Information Criterion (BIC). The plot of BIC versus the number of clusters (Fig. 3A) showed a clear decrease in BIC as K increased from 1 to 4. Beyond K = 4, the BIC values began to plateau and fluctuate, with a less pronounced rate of decline. This inflection point, or “elbow,” suggests that the most meaningful and robust genetic structure exists at K = 3 or K = 4. Given the strong differentiation observed at K = 3 and the clear separation of groups from earlier approaches, this was considered the optimal subpopulation. The scatter plot visualized this genetic structure by maximizing between-group variation and minimizing within-group variation. It divides the inbred lines into three distinct, non-overlapping clusters labeled as cluster one in red (911 inbred lines), two in blue (380 inbred lines), and three in green (146 inbred lines) (Fig. 3B). When the DAPC clustering results was integrated with grouping from ancestral admixture Q matrix, the first cluster having 911 inbred lines has 340 inbred lines characterized by mixed ancestry, cluster two with 380 inbred lines has 40 inbred lines characterized by mixed ancestry, while cluster three has no admix individual (Table S3). The density plot of the first discriminant function (Fig. 3C) offers an alternative view of the separation, displaying three distinct, non-overlapping peaks that match the three identified clusters. The large gaps between these peaks highlight significant genetic differentiation among these groups.

**Fig. 4:**
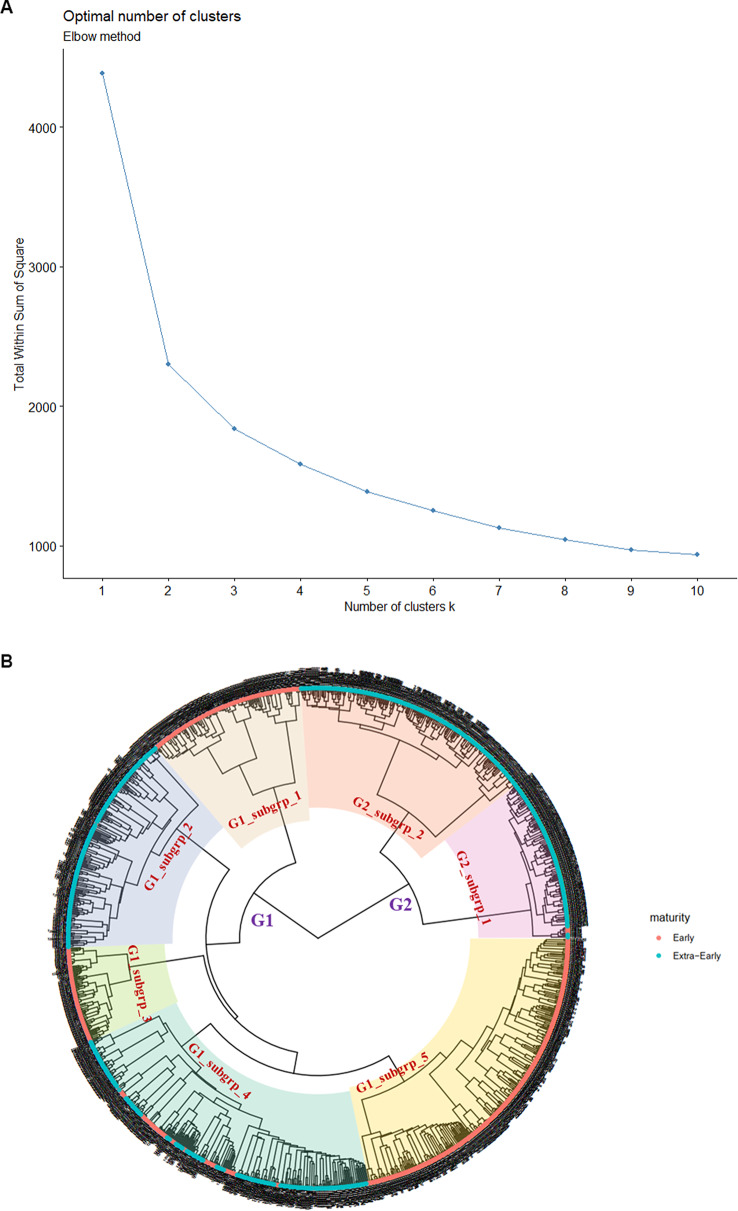
Phylogeny tree from phylogenetic analysis showing (**A**) optimum number of cluster at K = 2, (**B**) visualization of the two genetic groups and their respective subgroups characterized by maturity group. Red coloration indicates early maturing inbreds and cyan color indicates extra-early maturing inbreds

The phylogeny tree (phylogenetic analysis), which shows how inbred lines are related or distant from one another based on genetic distance, revealed slightly different results from those obtained by other methods. While PCA, DAPC, and ancestral admixture analysis indicated three groups, the optimal number of clusters for constructing the phylogeny tree was K = 2 (Fig. 4A), indicating two genetic groups. The first group contained a larger number of inbred lines (1062 inbred lines equivalence of 74%), while the second group had 375 inbred lines, equivalence of 26%. With this observation, the results from DAPC were integrated into the phylogenetic groups to see where the differences occur in inbred assignment, and it showed that the first group from the phylogenetic analysis comprised of the first and third group in the DAPC results (Table S3). While the second group was maintained across both methods. Upon adding the grouping from Q matrix (ancestral admixture), phylogenetic group one with 1062 inbred lines maintained the 340 inbred lines with mixed ancestry while group two also maintained 40 inbred lines as admix.

The characteristics of each phylogenetic group differ with respect to genetic distance (GD). For the first group, which included inbreds from both maturity groups, the average GD was 0.384, with a maximum GD of 0.490 between TZEI 2962 and TZEEQI 412, and a minimum GD of 0.002 between TZEIOR 444 and TZEIOR 443. The second group, characterized by the extra-early maturity inbred lines, had an average GD of 0.260, a maximum GD of 0.415 (between TZEEIOR 435 and TZEEI 421), and a minimum GD of 0.004 (between TZEEIOR 531 and TZEEIOR 431). Interestingly, several subgroups—five in group one and two in group two—existed within these two main genetic groups. As observed in previous analysis results, kernel color does not correlate with the main groups identified (Sup. Figure 3). However, maturity groups showed a strong alignment with the inferred genetic groups and further defined the characteristics of each subgroup. Among the subgroups in main group one, subgroups one, three, and five consisted solely of early-maturity inbred lines, while subgroup two comprised extra-early inbred lines (Sup. Figure 3 A). Although subgroup four included a mixture of inbreds from both maturity groups, extra-early maturing lines dominated. Main group two included only extra-early inbred lines across its subgroups, defining this group (Fig. 4B).

### Delineation and refinement of heterotic groups

**Fig. 5:**
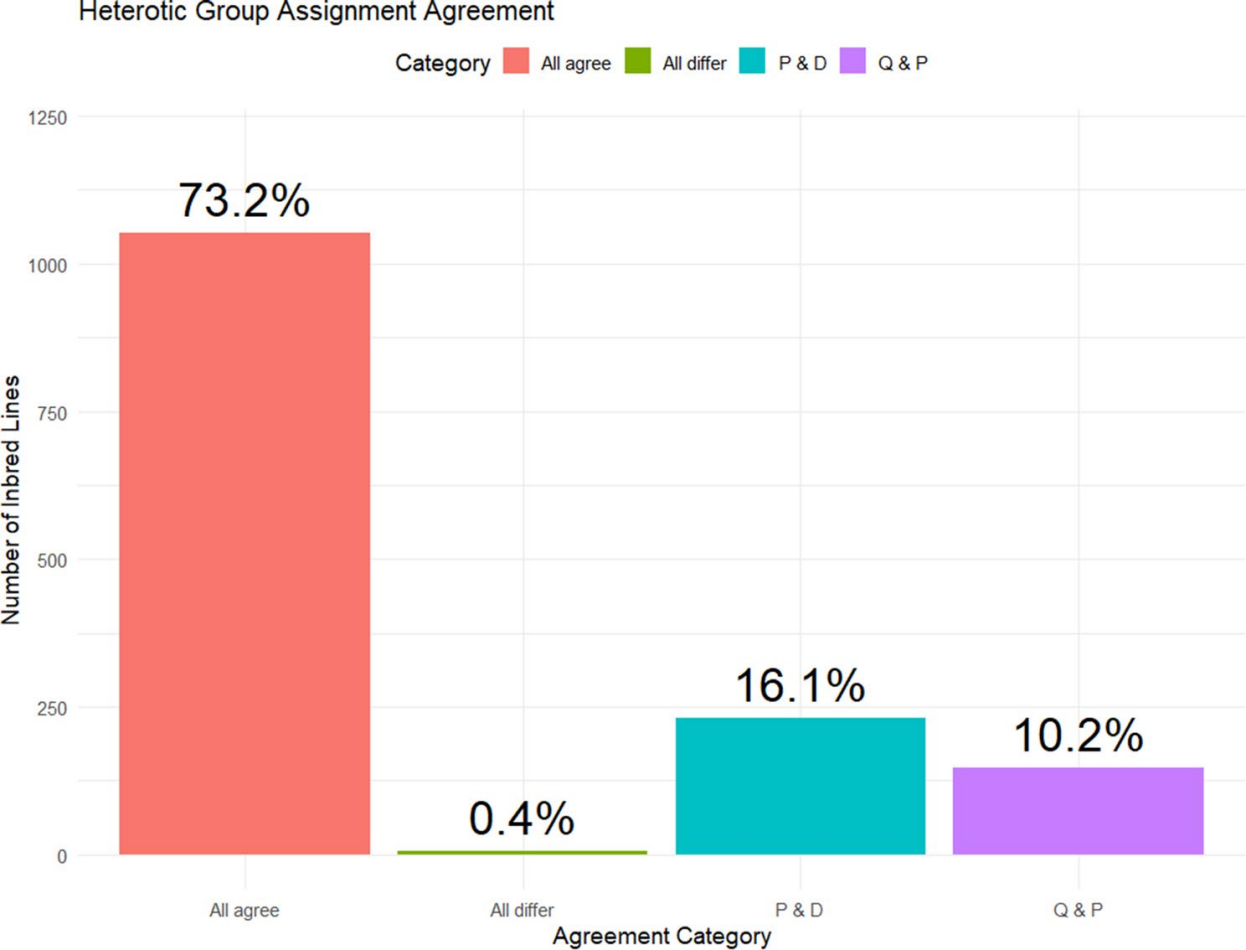
Bar plots showing the potential heterotic group assignment agreement among Q matrix from ancestral admixture analysis, discriminant analysis of principal component, and phylogenetic genetic analysis

To define and refine the heterotic groups of the 1,437 elite inbred lines, the grouping pattern obtained from DAPC, ancestral admixture Q matrix, and phylogenetic analysis were integrated for defining the final heterotic groups of the panel of elite inbred lines. Though DAPC and ancestral admixture Q matrix inferred three groups, phylogenetic analysis inferred two groups with the third group from DAPC being a subgroup in the larger (first) group from the phylogenetic analysis. For the purpose of identify how the three methods agree in assignment on inbred lines into distinct groups, we recoded the third group from the DAPC as a subgroup of the larger phylogenetic group and force-assigned all admixed individuals to the group where they had the highest membership probability (even if that probability was < 70%). An agreement of 73.2% was observed across the three methods while 26.8% accounted for assignments differing by at least one method (Fig. 5). To facilitate practical implementation in the breeding program, the inbred lines were assigned into two main heterotic groups, consistent with the phylogenetic analysis. This allow visualization and identification of possible heterotic subgroups within each of the inferred group. In addition, within each of the main group there are distinct subgroups characterized by maturity class (Table 3). Furthermore, the eight inbred testers showed average divergence ranging from 0.52 in TZEI 17 and TZEEI 79 to 0.54 in TZEIOR 164, TZEEIOR 197, and TZEEIOR 97, and TZEEI 58. Several other inbred lines showed these range of divergence as suitable potential testers. The best 10 were from main heterotic group one including TZEEIOR 410, TZEI 2962, TZEIORQ 26, TZEEIOR 205, TZEEIOR 345, TZEQ I74, TZEIORQ 45, TZEEIOR 122, TZEEIOR 127, and TZEEIOR 165 with average divergence varying from 0.55 to 0.63 (Table S3).

**Table 3:** Summary of pure and admixed inbred lines in the heterotic main and sub-groups

Main heterotic group	Heterotic sub-group	Number of pure inbred line	Number of admixed inbred line	Maturity group
**Group 1**	subgroup 1	146	0	Early
	subgroup 2	29	178	Extra-early dominates
	subgroup 3	86	3	Early
	subgroup 4	149	160	Extra-early dominates
	subgroup 5	311	0	Early
Total		**721**	**341**	
**Group 2**	subgroup 1	145	5	Extra-early dominates
	subgroup 2	191	34	Extra-early
Total		**336**	**39**	
Grand Total		1057	380	

### Validation of the putative heterotic groups

Owing to several distinct subgroups characterized by maturity class within the two main potential heterotic groups, hybrids developed from within and between these maturity-based subgroups and evaluated showed significant effects for all measured traits. The grain yield performance of hybrids from between-heterotic subgroups for extra-early averaged 5071 kg/ha with a range of 4501 kg/ha to 6185 kg/ha. For hybrids from within-heterotic subgroup for the same maturity class was 4097 kg/ha (range: 2530 kg/ha – 5450 kg/ha). For early maturity, the grain yield performance of hybrids from between-heterotic subgroups ranged from 5003 kg/ha to 7610 kg/ha with an average of 5753 kg/ha, while that of within-heterotic subgroups ranged from 3768 kg/ha to 5420 kg/ha with an average of 4635 kg/ha (Table S3 and S4).

**Fig. 6:**
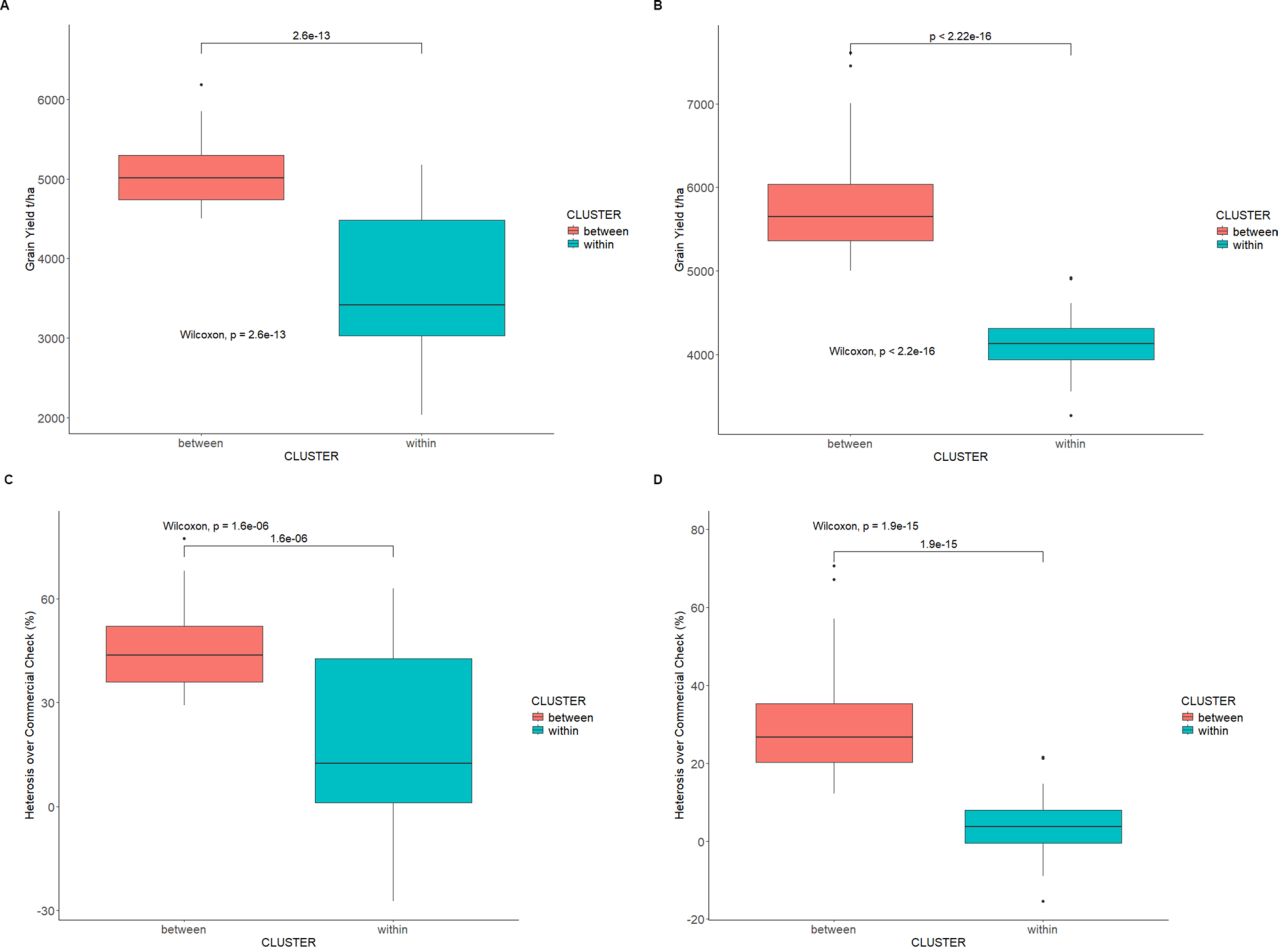
Comparison of grain yield performance of (**A**) between- and within-heterotic subgroups extra-early maturing maize hybrids, (**B**) between- and within-heterotic subgroups early maturing maize hybrids, (**C**) standard heterosis over best commercial check for between- and within-heterotic subgroups extra-early maturing maize hybrids, and (**D**) standard heterosis over best commercial check for between- and within-heterotic subgroups early maturing maize hybrids

Standard heterosis estimates from between-heterotic subgroups in extra-early maturity ranged from 29.17% to 77.50% with an average of 45.53%, while those of within-heterotic subgroups ranged from − 27.39% to 56.40% with an average of 16.22%. These estimates were derived from comparing the performance of the best extra-early check called Sosani (released and commercialized in Mali). The check mean performance was 3,485 kg/ha. In a similar manner, the standard heterosis estimates from hybrids between-heterotic subgroups in early maturity ranged from 12.15% to 70.74% with an average of 30.00% while those of within-heterotic subgroups ranged from − 15.46% to 21.60% with an average of 3.99%. These estimates were derived from comparing the performance of the best early check called SAMMAZ 41 (in Nigeria) / Tamalaka (in Mali) / CSIR-Denbea (in Ghana). The check mean performance was 4,457 kg/ha. The average performance of hybrids from between-heterotic subgroups consistently and significantly exceeded that of hybrids from within-heterotic subgroups (Fig. 6A and B, Table S4 and S5). In addition, the average standard heterosis estimate against the best commercial checks was significantly higher for hybrids from between subgroups than within heterotic subgroups (Fig. 6C and D, Table S4 and S5).

## Discussion

### Genetic diversity

The utility of a genomic resource in tropical maize breeding lies in its power to inform not only selection but also strategic hybrid development. The SNP panel used in this study was highly informative, with average MAF (0.265) and PIC (0.328) values supporting its capacity to detect diversity and differentiate among lines [35, 36]. The low observed heterozygosity (0.032) among S_7_ lines reflects successful inbreeding, making these lines suitable candidates for hybrid formation and inbred extraction [37]. Conversely, the high expected heterozygosity (0.315) across the panel suggests broad allelic diversity, crucial for establishing heterotic groups and supporting long-term genetic gain.

### Genetic structure and primary drivers

Population structure analysis revealed that maturity group, rather than kernel color, is the main driver of genetic differentiation. This is consistent with IITA’s breeding pipelines, where early and extra-early maturity classes are developed as separate breeding streams to enhance adaptation to the diverse agro-ecologies of West and Central Africa [38]. This finding aligns with the fact that several yellow and orange color inbred lines share similar ancestor/pedigrees. Additionally, the findings hold significant practical utility for IITA’s maize breeding pipelines, as it implies that yellow and orange kernel maize can be strategically grouped based on maturity and heterotic patterns, rather than requiring separate, independent heterotic groups defined solely by color. This simplifies the breeding scheme and maximizes the genetic pool size for selection within the key maturity classes. Genetically, this non-correspondence suggests that the kernel color traits (such as the provitamin A genes responsible for the orange phenotype) were introduced and fixed relatively recently through targeted introgression, resulting in the genetic architecture of color being largely decoupled from the deeper evolutionary divergence driven by maturity. This is a crucial advantage for nutritional breeding as it allows breeders to simultaneously select for high provitamin A content and maximize heterosis using the maturity-based G1 × G2 pool structure, without the need to maintain distinct color-based heterotic pools. This will accelerate the deployment of biofortified hybrids.

### Contrasting clustering methods and definitive heterotic group

Through PCA, admixture analysis, DAPC (K = 3), and phylogenetic reconstruction, two major heterotic groups emerged with maturity-specific subgroups nested within each group. It is noteworthy that the optimal cluster solutions provided K = 3 (DAPC, Admixture) and K = 2 (Phylogeny) results. This discrepancy reflects the difference in algorithms. Admixture and DAPC are sensitive to fine-scale, recent substructure, while Phylogenetic analysis identifies the deepest evolutionary divergence. For breeding utility, the K = 2 split was adopted as the definitive heterotic break, maximizing inter-pool heterosis and aligning with standard two-pool recurrent selection, with the K = 3 structure providing useful information for subgroup refinement. This nested structure is not only biologically relevant but also practically valuable for hybrid development [16, 37]. Our finding that the IITA-MIP yellow/orange short-duration maturing germplasm is best partitioned into two major heterotic groups, which are further refined by several distinct subgroups, is consistent with global efforts to rationalize maize breeding pools. The use of SNP markers to define these groups aligns with the methodology successfully employed in other major breeding programs. Arora et al. [39] working with tropical maize germplasm in India similarly demonstrated the high concordance between marker-based clustering and empirical combining ability estimates. Our results reinforce their conclusion regarding the utility of SNP-based grouping for tropical materials, particularly given the consistency of our between-subgroup crosses showing superior performance, which validates the heterotic-subgroup partitioning. Furthermore, the need to partition major groups into subgroups (G1S1, G1S2, etc.) is supported by findings in other tropical environments. Badu-Apraku et al. [16], focusing on West African early- and extra-early-maturing maize germplasm pool similar to ours, found that sub-grouping was critical for maximizing hybrid vigor. Finally, while our germplasm is strictly tropical, the level of heterosis observed in our optimal inter-group crosses (∼30% − 70% standard heterosis) is highly competitive and reflects the strong genetic divergence that has historically driven success in temperate breeding, such as the classic Stiff Stalk × Lancaster split. Ma et al. [40] used SNP markers to highlight that while maize is highly structured, the genetic divergence between pools remains the principal factor governing heterosis. Our work confirms that this principle holds true, and that the identified IITA-MIP groups offer a robust, high-yielding pattern for hybrid development in Africa.

### Application to hybrid strategy

These findings provide a good framework for implementing single and three-way hybrid strategies, which are integral to IITA’s product development pipeline. Of these two hybrid types, three-way hybrids offer greater stability and resilience than single crosses, especially under low-input or stress-prone tropical conditions [6, 8]. The observed genetic subgroup within the two main heterotic groups provide a rational basis for forming robust three-way hybrids. The subgroup is instrumental in this process by allowing for parental refinement. It ensures that while the main heterotic break (G1 × G2) maximizes final grain yield heterosis, the subgroups perfect the crosses to maximize F1 seed yield and ensure successful, synchronized pollination, which is a critical factor for success in variable tropical environments. To achieve this, elite single crosses can be derived from intra-group parental lines (e.g. early × early or extra-early × extra-early), which are then crossed to testers or lines from the opposing heterotic group to form high-performing three-way hybrids. This approach capitalizes on both within-group adaptation and between-group heterosis. In another approach, a high-yielding early maturity hybrid could be developed by first selecting a broadly adapted and high-performing intra-early single cross (e.g. G2_SG1 × G2_SG3), which is then crossed to a contrasting extra-early line from G1 (SG1 or SG2). This design ensures both genetic distance (to maximize heterosis) and agronomic compatibility (to match environmental adaptation and flowering time), which is particularly important for Sub-Saharan Africa variable growing conditions.

### Managing residual germplasm and sustained genetic gain

The genetic distance matrix supports this approach. Lines within clusters showed close relatedness (minimum GD = 0.002), while those between clusters showed substantial divergence (maximum = 0.49). The practical utility of this genomic framework is further realized in the management of the inbred germplasm not clearly assigned to the major pools. Our rigorous grouping methods identified a specific, manageable subset of 26% of the germplasm as admixed (lines with mixed ancestry below the membership threshold). This manageable proportion has two direct, quantified impacts on our operational strategy. These include higher breeding efficiency and targeted source population. With 74% of the lines now clearly assigned to the designated genomic heterotic groups, our program can immediately execute highly efficient, targeted inter-group crosses (G1 × G2), maximizing the frequency of high-yielding 3-way hybrids. Additionally, the 26% admixed pool that observed will form the primary source population for continuous genetic improvement. These lines, possessing a combination of alleles from the ancestral pools, will be subjected to intensive selection and inbreeding efforts to achieve some objectives that may not be met by crossing within the established diverge pools. Specifically, targeted selection in this pool is essential to break unfavorable genetic correlations (between high yield and resistance/tolerance biotic and abiotic stresses) that slow genetic gain in the breeding program.

### Empirical validation

The high agreement among DAPC, admixture, and phylogenetic assignments provides confidence to the proposed heterotic groupings. Such consistency justifies their adoption into IITA’s structured breeding pipelines. More importantly, hybrid performance data validated the utility of these molecular-based groupings. Crosses between distinct maturity-based subgroups consistently outperformed within-group hybrids, yielding up to 1,100 kg/ha more in early maturity lines and nearly 1,000 kg/ha in extra-early maturity lines. Standard heterosis was also significantly higher in between-subgroup crosses (45.5% and 30%) compared to within-subgroup crosses (16.2% and 4%), reinforcing the biological validity of the defined heterotic groups. These results demonstrate that the genetic groupings derived from molecular markers are not just theoretical constructs but translate into real gains in hybrid performance. Beyond hybrid development for farmers’ benefit, the structured genetic diversity in heterotic groupings supports sustained inbred line improvement within IITA’s breeding pipelines. By selecting within each subgroup, new breeding populations can be advanced through pedigree or recurrent selection to yield elite lines tailored to early or extra-early maturity windows, while retaining divergence for heterotic exploitation. Divergent lines identified as potential testers can expand the tester panel after validation through testcross performance or Diallel analysis. Such testers are critical in discriminating new inbreds and assigning them to appropriate heterotic groups [41, 42], especially within three-way hybrid development frameworks that rely on complementary heterotic pools for top and base parents [41, 42].

In summary, this study not only elucidates the genetic structure of a diverse tropical maize panel but also presents actionable insights directly applicable to IITA’s breeding strategy. By anchoring heterotic grouping in maturity-based genomic substructure, maize breeders can optimize both single and three-way hybrid design. The integration of molecular data with field performance underpins a precision breeding framework that enhances the efficiency of hybrid development and the extraction of superior inbred lines tailored for sub-Saharan Africa’s maize production environments.

### Integrating genomic selection and future strategy

The proven utility of the defined heterotic structure, as validated by superior field performance and high standard heterosis, provides a powerful framework for the immediate integration of genomic selection (GS) into the IITA breeding pipeline. By partitioning the germplasm into distinct, less admixed pools (G1 and G2), the GS model’s predictive accuracy can be significantly enhanced. This structure allows for the adoption of reciprocal genomic selection (RGS) for hybrid prediction. The goal of RGS is to simultaneously improve the general combining ability (GCA) of both heterotic pools (G1 and G2) for hybrid performance. We can achieve this by creating group-specific GS models, trained on the performance of the G1 × G2 hybrids. This will allow our breeders to predict the hybrid performance of new lines before costly field trials. Furthermore, implementing GS within these defined subgroups creates training populations that are smaller and more closely related, sharing higher levels of linkage disequilibrium (LD) with the selection candidates. This LD-based increase in model accuracy for within-group GCA and between-group Specific Combining Ability (SCA) prediction. This will allow for faster cycle times and accelerated selection for complex traits like grain yield and resilience to key stress factors. This strategic use of RGS will complement the validated heterotic structure and ensure maximum return on investment in the breakthrough hybrid-breeding program.

### Study limitations and future research directions

While the 3,305 SNPs provided high informativeness (average MAF = 0.265) and sufficient power to resolve the primary heterotic structure, the mid-density nature of the DArTag panel may have a limited capacity to capture rare variants or small-effect quantitative trait loci (QTLs). We recommend future studies utilizing Genotyping-by-Sequencing (GBS) or whole-genome sequencing to fully saturate the genome and ensure the maximum capture of allelic diversity, particularly within the 26% admixed germplasm. Additionally, we conducted the empirical validation of our genomic heterotic groups at a single, high-potential environment (Ikenne). While this site is excellent for accurately estimating maximum heterosis potential, the superior performance of our inter-group hybrids must be validated across diverse environments. We acknowledge that genotype by environment interactions are a significant factor in tropical maize. Therefore, we recommend that the newly defined genomic heterotic groups further evaluated at multi-locations for stability.

## Conclusion

This study employed multiple complementary methods to examine the genetic diversity, population structure, and heterotic grouping of 1,437 elite yellow and orange maize inbred lines from early and extra-early maturity groups. The SNP markers used showed high levels of polymorphism and expected heterozygosity, indicating ample genetic variation. Population structure analysis through PCA, ancestral admixture, and DAPC consistently identified three major genetic clusters, mainly influenced by maturity group but not kernel color. Phylogenetic reconstruction revealed two primary clusters that more clearly corresponded to maturity classes and allowed further division into meaningful subgroups. Despite slight differences among these methods, we observed a strong agreement (73%) with group assignment. This consensus led to a biologically meaningful and practically useful classification of the inbred lines into two main heterotic groups, each further divided into subgroups reflecting maturity classes. From these subgroups, ten inbred lines with distinct maturity backgrounds were identified as potential testers. Validation revealed that hybrids derived from crosses between different heterotic subgroups consistently exhibited superior yield performance and higher heterosis estimates. These refined groupings establish a strong foundation for strategic hybrid development, effective parental selection, and enhanced heterosis exploitation in tropical extra-early and early maize breeding programs.

## Supplementary Information

Below is the link to the electronic supplementary material.

Supplementary Material 1

Supplementary Material 2

## Data Availability

The maize DArTag SNP genotyping study is registered under NCBI BioProject PRJNA1391357. This BioProject (PRJNA1391357), initially under embargo has been released for public access on January 7, 2026. The BioSample accessions SAMN54259255–SAMN54260204 and SAMN54260225–SAMN54260711 correspond to leaf-derived DNA from maize inbred lines used for SNP genotyping. The processed DArTag SNP genotype matrix supporting the findings of this study is attached as supplementary file and also publicly available via Figshare at. [10.6084/m9.figshare.29966027.v1].

## Abbreviations

SSA: Sub-Saharan Africa
IITA: International Institute of Tropical Agriculture
MIP: Maize Improvement Program
BIC: Bayesian Information Criterion
DAPC: Discriminant Analysis of Principal Component
GD: Genetic Distance
G1 and 2: Heterotic Group one and two
SG1, 2, 3: Heterotic sub-group one, two, and three
MAF: Minor Allele Frequency
OH and EH: Observed and Expected Heterozygosity
PIC: Polymorphism Information Contents
