# Supplementary material for: Genomic-assisted refinement of heterotic groups in short-duration maturing tropical yellow and orange maize inbred lines
Source: BMC Agric. 2026 Mar 4;2(1):9. doi: 10.1186/s44399-026-00032-2 (PMC12980660; doi:10.1186/s44399-026-00032-2)
Supplement: Supplementary file 1 — Supplementary Material 1 [file 44399_2026_32_MOESM1_ESM.docx]

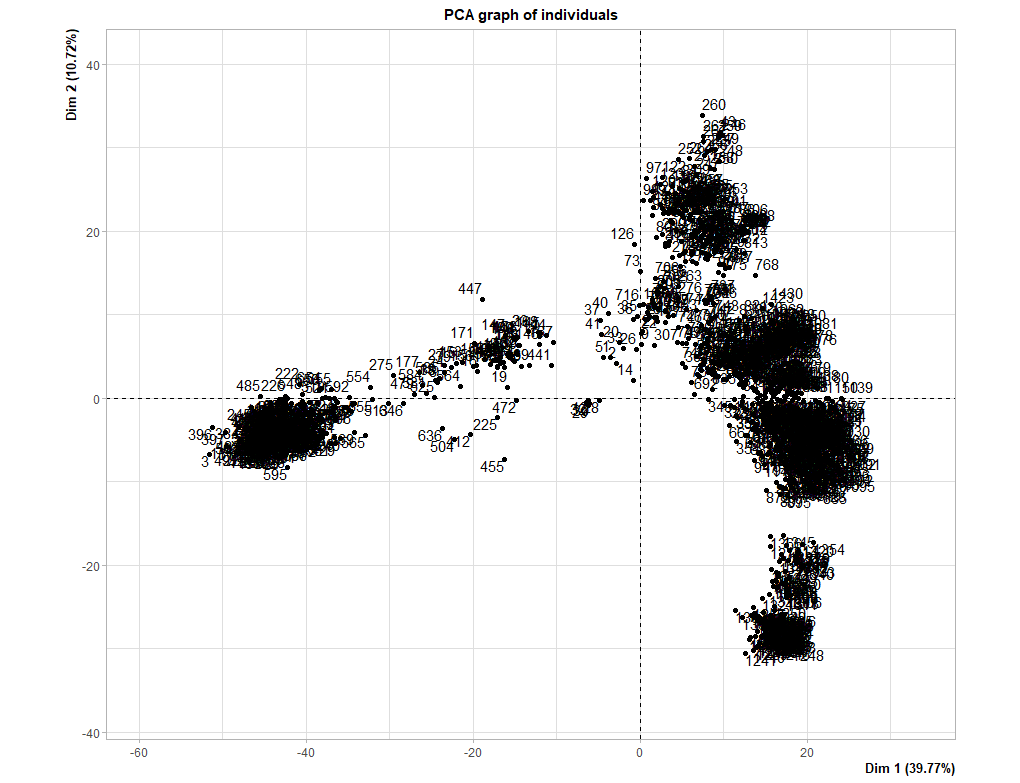


Fig. S1. Visualization of population stratification using principal component analysis.

Fig. S2. Visualization of population stratification overlaid by (A) kernel color and (B) source populations (C) maturity group from principal coordinate analysis.


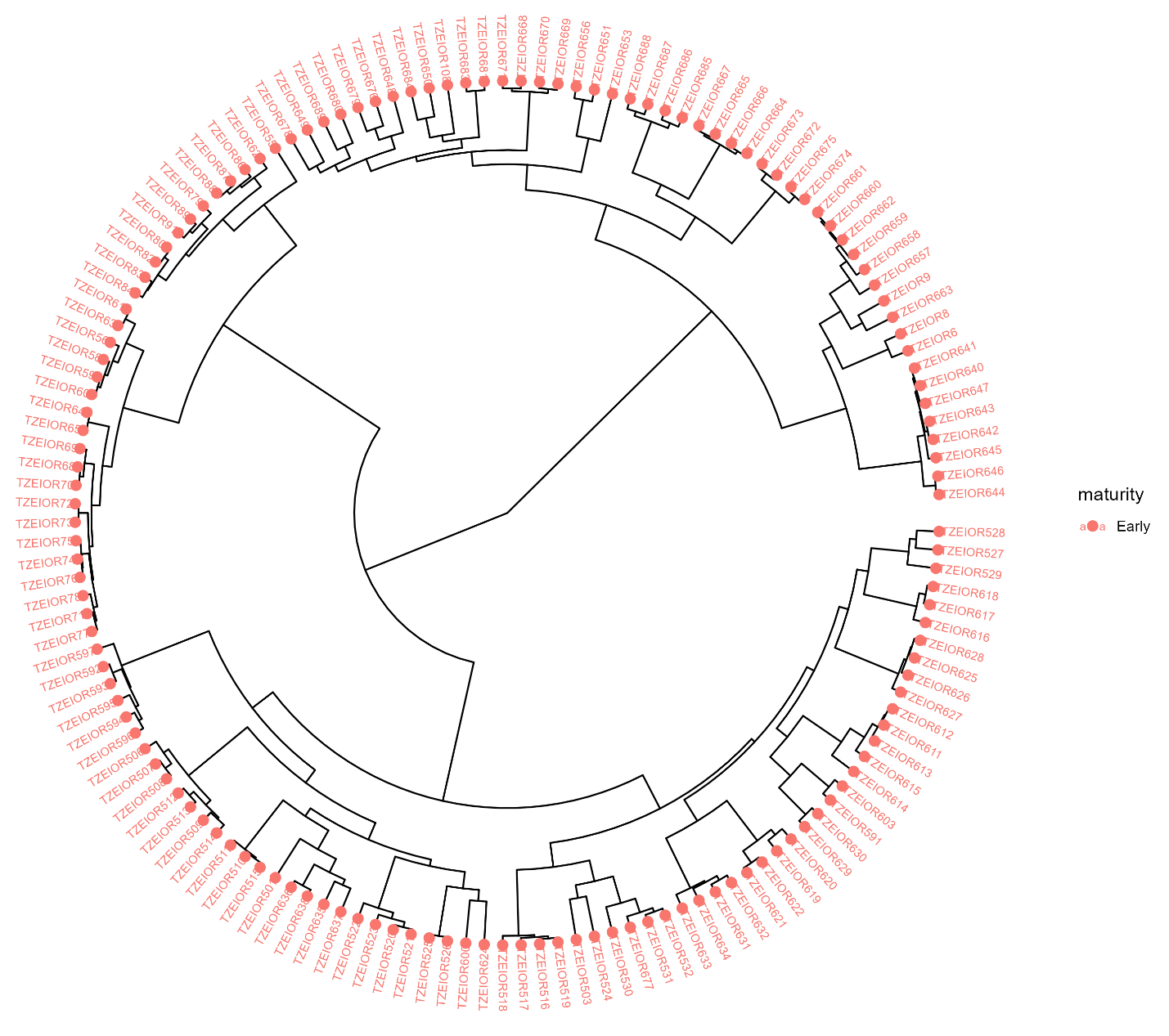


Fig. S3A. Phylogeny tree showing the inbred lines in the main heterotic group one subgroup one consisting of only early maturing class inbred lines. Red coloration indicate early maturing inbred lines.


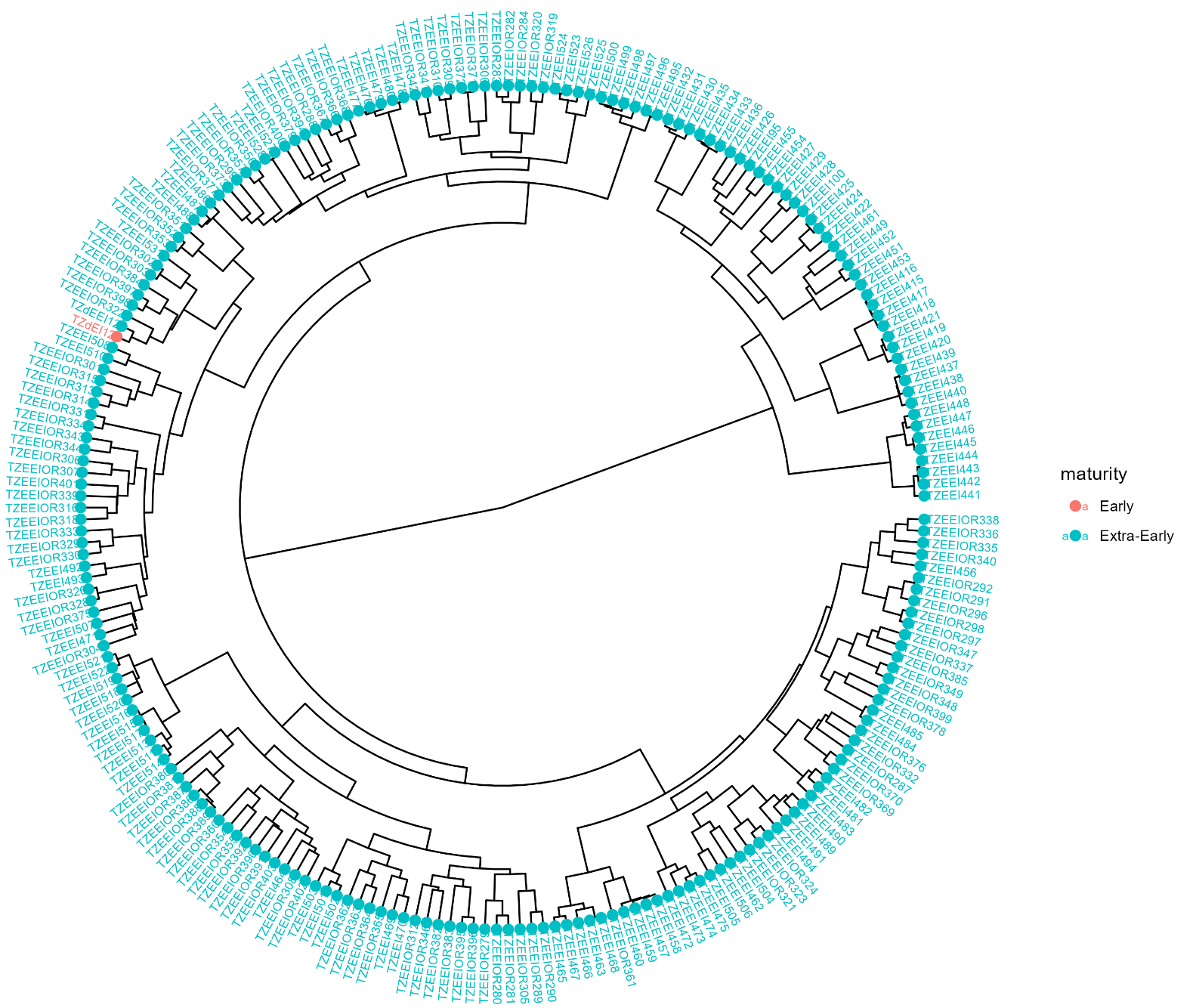


Fig. S3B. Phylogeny tree showing the inbred lines in the main heterotic group one subgroup two dominated by extra-early maturing class inbred lines. Red coloration indicate early maturing inbred lines while cyan coloration indicates extra-early inbred lines.


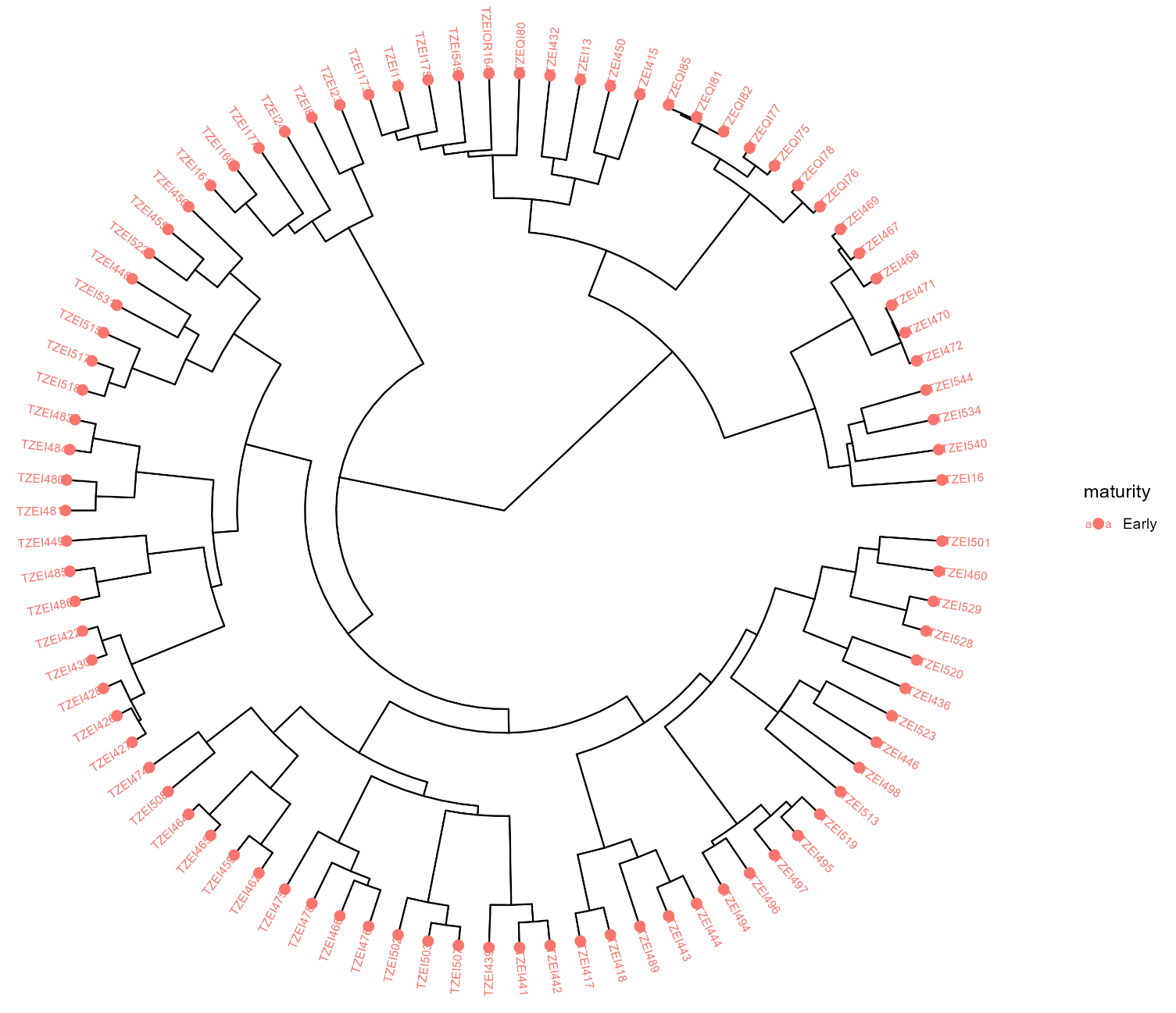


Fig. S3C. Phylogeny tree showing the inbred lines in the main heterotic group one subgroup three comprising only early maturing class of inbred lines. Red coloration indicate early maturing inbred lines.


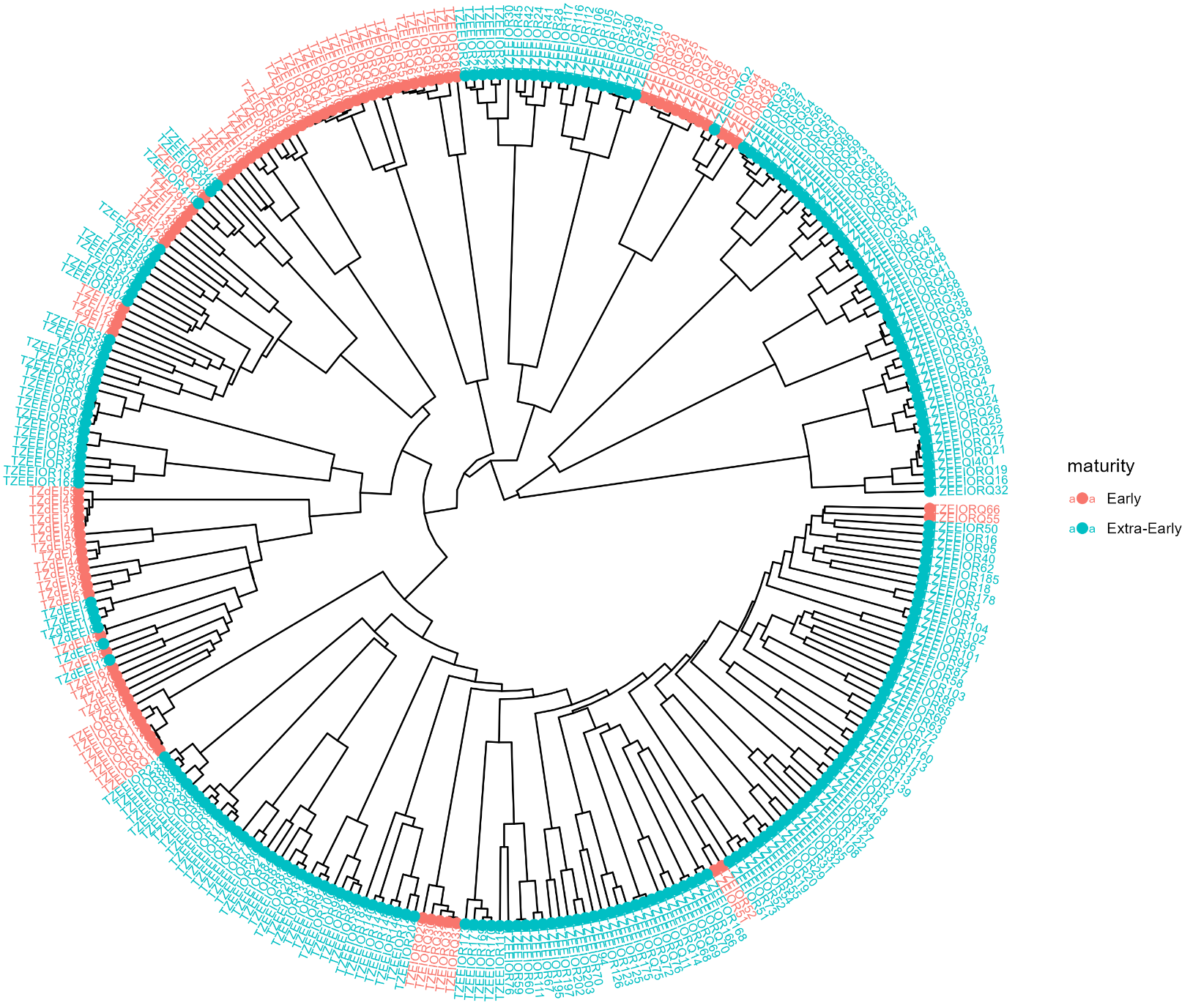


Fig. S3D. Phylogeny tree showing the inbred lines in the main heterotic group one subgroup four combining both extra-early and early maturing classes but largely dominated by the extra-earlyclass of inbred lines. Red coloration indicate early maturing inbred lines while cyan coloration indicates extra-early inbred lines.


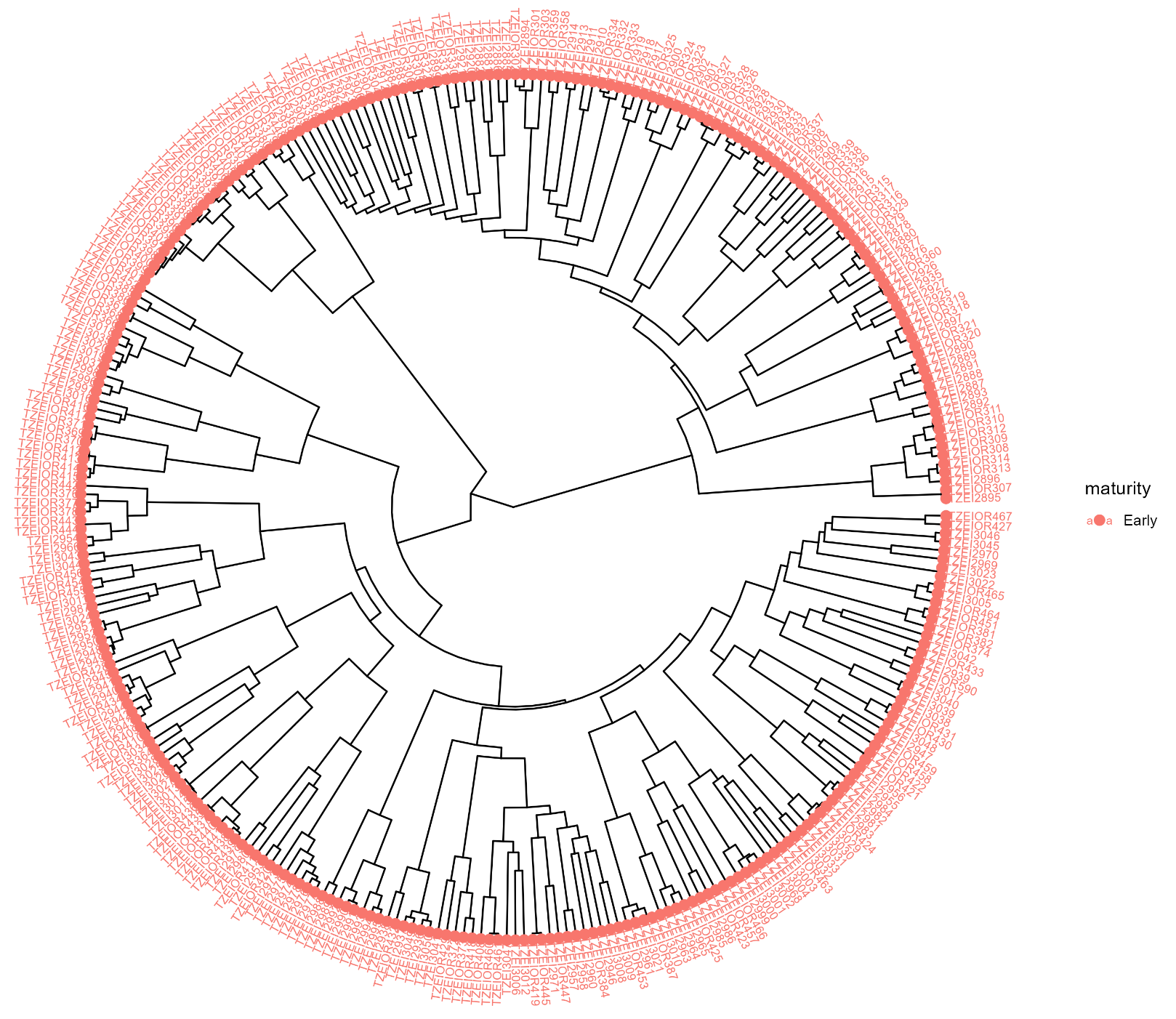


Fig. S3E. Phylogeny tree showing the inbred lines in the main heterotic group one subgroup five consisting of only early maturing class of inbred lines. Red coloration indicate early maturing inbred lines.


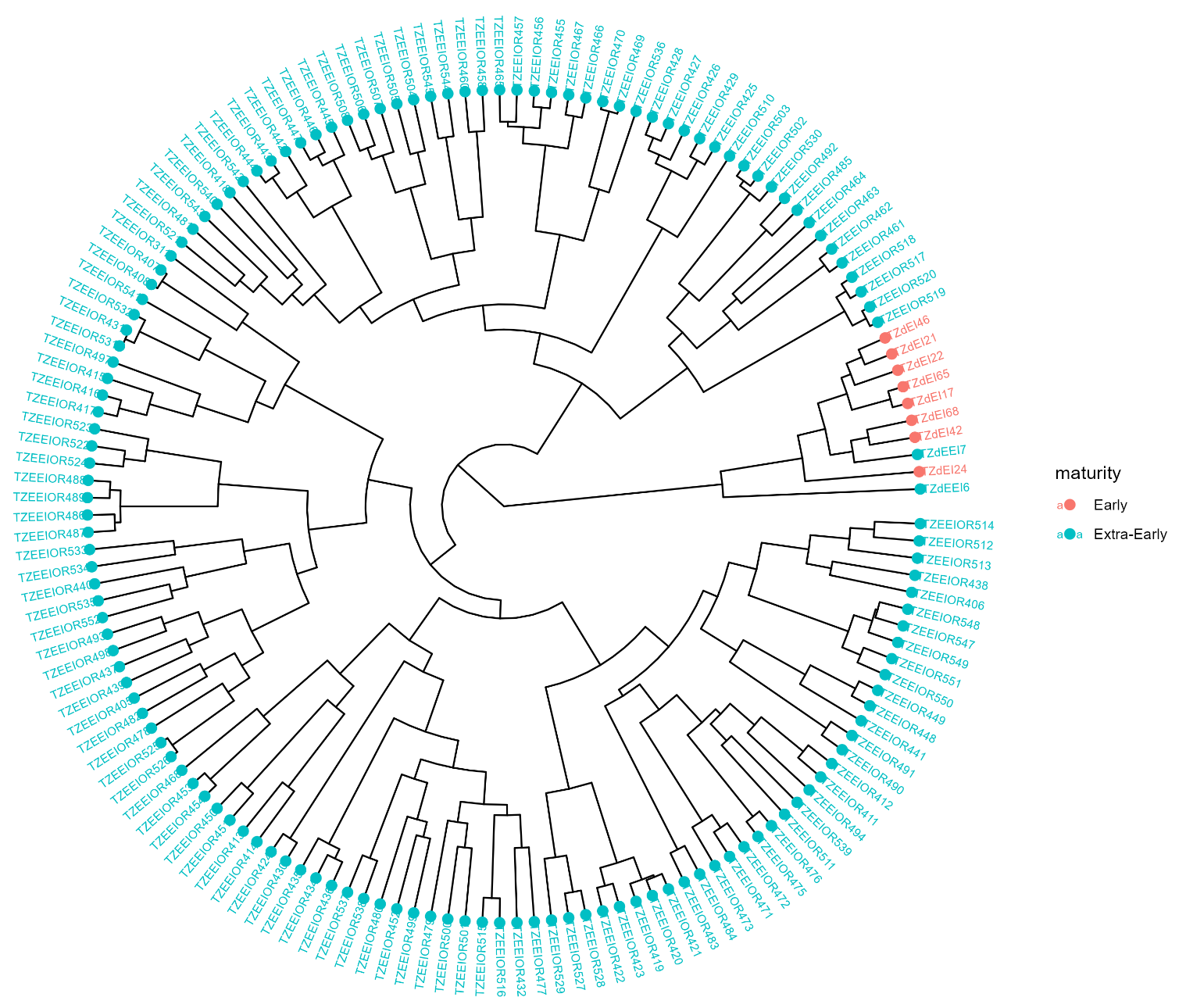


Fig. S3F. Phylogeny tree showing the inbred lines in the main heterotic group two subgroup one combining both extra-early and early maturing classes but largely dominated by the extra-earlyclass of inbred lines. Red coloration indicate early maturing inbred lines while cyan coloration indicates extra-early inbred lines.


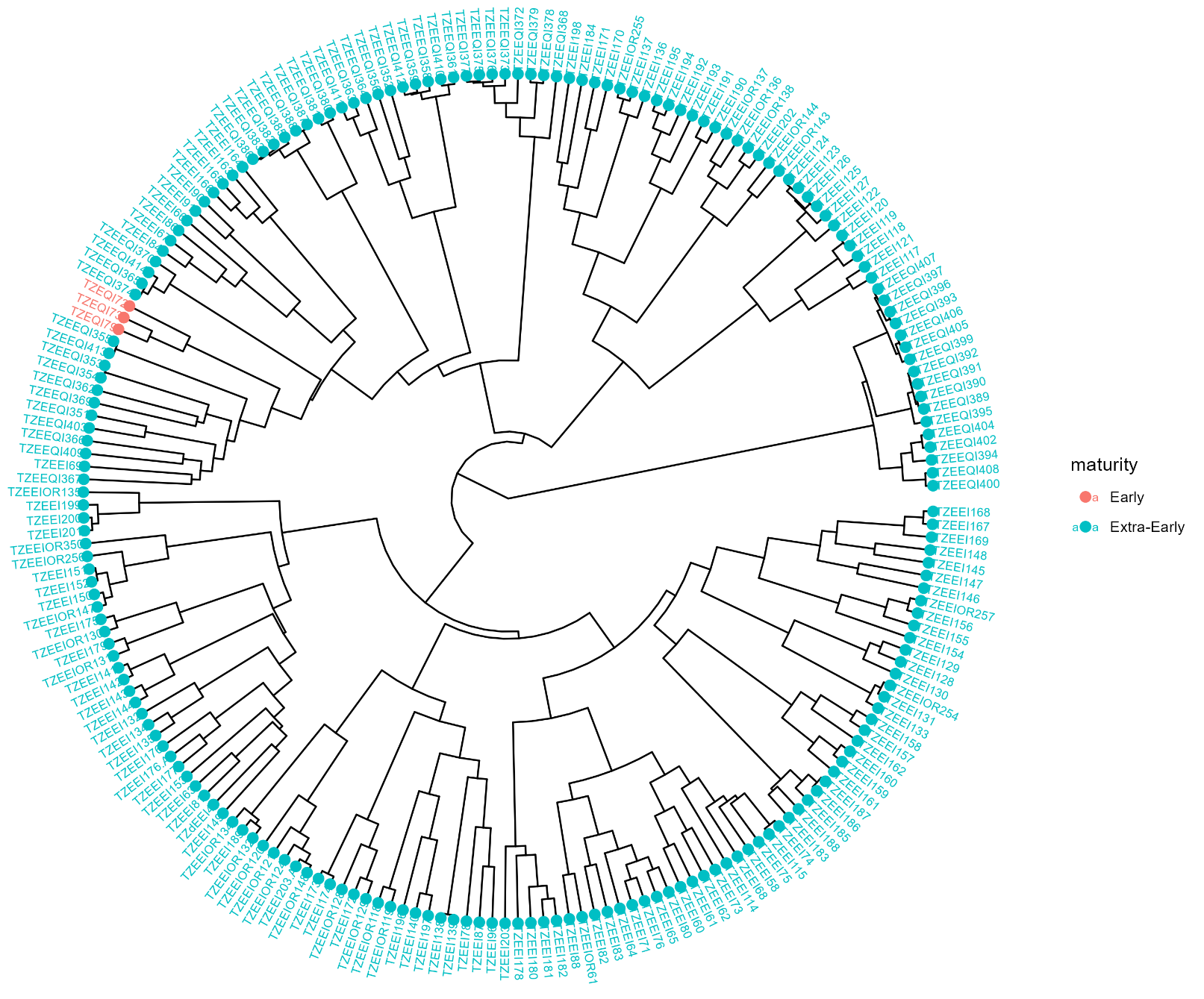


Fig. S3G. Phylogeny tree showing the inbred lines in the main heterotic group two subgroup two combining both extra-early and early maturing classes but largely dominated by the extra-earlyclass of inbred lines. Red coloration indicate early maturing inbred lines while cyan coloration indicates extra-early inbred lines.
